# Interface Design Principles for High‐Performance Organic Semiconductor Devices

**DOI:** 10.1002/advs.201500024

**Published:** 2015-03-23

**Authors:** Wanyi Nie, Gautam Gupta, Brian K. Crone, Feilong Liu, Darryl L. Smith, P. Paul Ruden, Cheng‐Yu Kuo, Hsinhan Tsai, Hsing‐Lin Wang, Hao Li, Sergei Tretiak, Aditya D. Mohite

**Affiliations:** ^1^ Material Synthesis and Integrated DevicesMPA‐11, Los Alamos National LaboratoryLos AlamosNM87545USA; ^2^ Department of Electrical and Computer EngineeringUniversity of MinnesotaMinneapolisMN55455USA; ^3^ Physics of Condensed Matter and Complex Systems DivisionLos Alamos National LaboratoryLos AlamosNM87545USA; ^4^ Physical Chemistry and Applied SpectroscopyChemistry DivisionLos Alamos National LaboratoryLos AlamosNM87545USA

**Keywords:** charge transfer state, interface, organic solar cell, recombination

## Abstract

**Precise manipulation of organic donor‐acceptor interfaces using spacer layers** is demonstrated to suppress interface recombination in an organic photo­voltaic device. These strategies lead to a dramatic improvement in a model bilayer system and bulk‐heterojunction system. These interface strategies are applicable to a wide variety of donor–acceptor systems, making them both fundamentally interesting and technologically relevant for achieving high efficiency organic electronic devices.

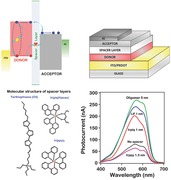

Interfaces are crucial for controlling charge and energy transport of carriers in organic electronics. In organic photovoltaics (OPV), the photocurrent generation occurs via three basic photophysical processes: (a) Exciton generation in the donor (or acceptor) region and migration to a donor–acceptor interface; (b) charge transfer (CT) state formation from an exciton at the interface; and (c) CT state dissociation followed by charge collection at the device contacts.^1^ These processes are often expressed as recombination rates as illustrated in **Figure** **1**a: (i) exciton generation and migration to the interface, which competes with exciton recombination (*K*_ER_); (ii) exciton dissociation to CT state (*K*_ED_); (iii) CT state dissociation (or charge separation rate; *K*_CS_), which competes with CT state recombination (*K*_CTR_). Therefore, controlling and manipulating these rates is critical for achieving maximum power conversion efficiency (PCE).^2^, ^3^ An efficient OPV device mandates that exciton dissociation to form the CT state (hole on donor and electron on acceptor) occurs faster than exciton recombination and that charge separation occurs at a rate faster than CT state recombination.^4^, ^5^ Recent reviews and high impact publications have elucidated that a long‐standing scientific challenge for achieving high efficiency OPVs is the prevention of CT state recombination at the donor–acceptor interface.^3^, ^6^, ^7^ OPVs based on bulk heterojunction (BHJ) architectures have been extensively studied over the past decade but the exact role of process parameters and structural features on recombination rates and mechanisms that suppress charge transfer remains ambiguous.^5^, ^8^ These reports have motivated studies on understanding interface charge transport dynamics, however critical questions such as suppression of CT state recombination, mechanism of charge separation, and role of interface microstructure at the donor–acceptor interface remains a major scientific challenge for the organic electronics community.

**Figure 1:**
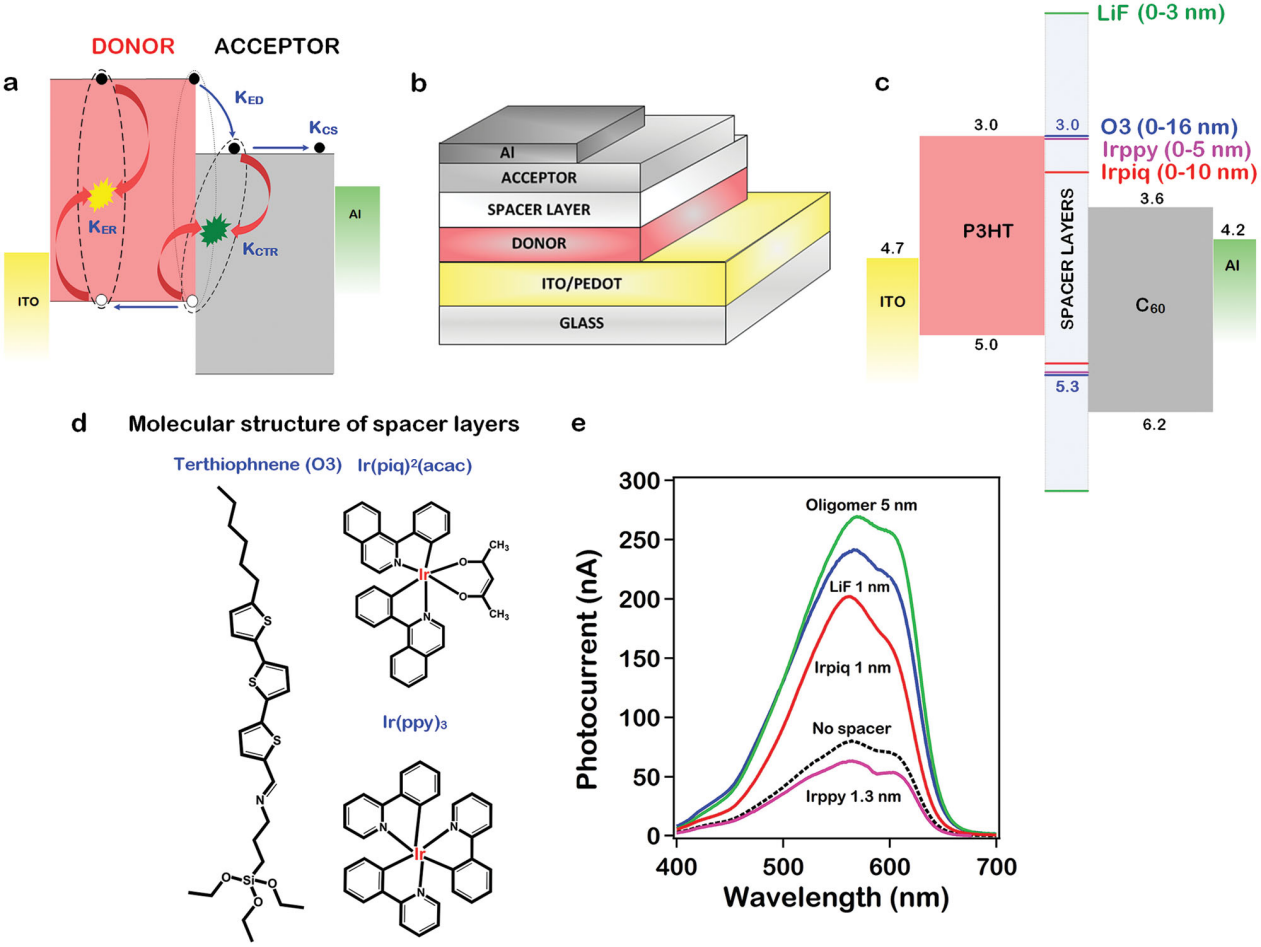
a) Photophysical processes and corresponding generation/dissociation/recombination rates in organic solar cells during device operation. b) Model bilayer device architecture. c) Strategies used in this study for inserting spacer layers at the P3HT/C_60_ interface and the energy level alignment of P3HT (donor) and C_60_ (acceptor) with respect to spacer levels. d) Molecular structures of O3, Irpiq, and Irppy spacer layers. e) Photocurrent versus excitation wavelength measured under short‐circuit conditions without (dashed black curve) and with (solid color curves) spacer layers. Peaks in the photocurrent spectra match well with the absorbance of P3HT (Figure S1, Supporting Information).

Here, we demonstrate three independent interface modification strategies, applicable to a broad class of donor–acceptor systems that dramatically suppresses the interface CT state recombination. We apply these strategies to a model bilayer OPV device that consists of ≈12 nm thick donor material [regio‐regular Poly(3‐hexylthiophene‐2, 5‐diyl) (P3HT)] and 30 nm of the acceptor material fullerene (C_60_). We modified the donor–acceptor interface between these two materials by adding the following spacer layers: (a) lithium fluoride (LiF); (b) terthiophene‐derivative (O3); metal organic complexes (c) (bis(1‐phenylisoquinoline)‐(acetylacetonate) iridium (III) (Irpiq); and (d) tris(2‐phenylpyridine)iridium (Irppy). For LiF, O3, and Irpiq spacer layers, we observe a dramatic ≈200%–350% increase in photocurrent and open circuit voltage (*V*_OC_), which translates to a ≈2–5 times increase in the overall PCE for the model bilayer device. Our photocurrent measurements indicate that a unique charge separation mechanism is responsible for the suppression of the CT state recombination for each spacer layer. We demonstrate that these proof‐of‐principle interface modification strategies can be applied successfully to practical OPV device architectures such as the BHJ, which dramatically enhances the overall PCE from ≈4.1% to up to 7.25%. These strategies can potentially serve as design principles for tailoring the recombination rates in donor–acceptor‐based optoelectronic devices and bring about an experimental protocol for achieving high efficiency OPVs and rejuvenate the nascent field of organic electronic devices.

Fundamental charge transport processes in OPVs and recombination rates associated with each process are depicted in Figure 1a. Figure 1b–d illustrate the model bilayer OPV device structure, strategies for interface modification using different spacer layers with varying energy levels and their molecular structure, respectively. The relevant energy levels were obtained from existing experimental data,^9^ cyclic voltammetry (see Figure S3 and Table S1, Supporting Information, and quantum chemical calculations described in Tables 3 and 4, Supporting Information). The photocurrent spectra were measured under short‐circuit conditions without (black dotted curve) and with (colored curves) the presence of thin interface modification layers (Figure 1e). A dramatic increase in the photocurrent of about 200–350% was observed for all the spacer layers (with the exception of Irppy) when inserted at the P3HT/C_60_ interface. Similar increase in the photocurrent was also observed for two other donor–acceptor polymer systems, (Tetracene/C_60_)^10^ and MtData/BPhen (Figure S4, Supporting Information). These results clearly suggest that CT state recombination can be suppressed in an organic polymer system by modifying the donor–acceptor interfaces with a few nanometers of a specifically designed functional interface spacer layer. (Detailed characterizations of bilayer absolute absorption and device internal quantum efficiency are discussed in Figure S5, Supporting Information.)

We use a previously established device model^11^ to interpret the experimental measurements and predict trends arising from the competition of the underlying interface recombination rates described in Figure 1a. The model establishes a clear link between the microscopic recombination rates at the interface (governed by structure dependent kinetic coefficients) and the macroscopic device performance, as characterized by the short‐circuit current, the open circuit voltage, and the PCE. By comparing experimental results and model calculations for bilayer devices that differ only in their microscopic interface structure, we demonstrate that it is indeed the interface structure through its impact on the CT state and its formation and recombination rates that controls the overall performance. (See Section 7, Supporting Information and Figure S7, Supporting Information, for description of device model.)

We describe in **Figure** **2**a–d the measured thickness‐dependent photocurrent spectra for devices with: (a) LiF, (b) O3, (c) Irpiq, and (d) Irppy spacer layers with the goal of investigating the underlying mechanism that allows us to control the recombination rates at the donor–acceptor interface. For the LiF spacer layer, the photocurrent increases with increasing layer thickness reaching a value of ≈300% of that for a device without a spacer layer, at a layer thickness of 1.0 nm, and subsequently decreases for thicker layers, eventually becoming smaller than for the device with no space layer. In sharp contrast, photocurrent for the O3 spacer layers keeps increasing up to a thickness of 5 nm after which it saturates at about ≈350% of the peak photocurrent and decreases for thicker layers. For the Irpiq spacer layers, the photocurrent increases with increasing layer thickness reaching a value of ≈250% of that for a device without a spacer layer, at a layer thickness of 1.0 nm. The photocurrent then decreases with increasing thickness with a decay rate that is intermediate than that of LiF or O3. However, the photocurrent for the Irppy spacer layers monotonically decreases with increasing spacer layer thickness. The thickness dependence of the photocurrent for the case of LiF, O3, and the metal organic spacer layers Irpiq and Irppy clearly suggests that different mechanisms are involved in the suppression of the CT recombination. Figure 2e schematically illustrates the relevant energy levels for the various spacer layers obtained from existing experimental data, cyclic voltammetry (see Figure S4, Supporting Information) and quantum chemical calculations.

**Figure 2:**
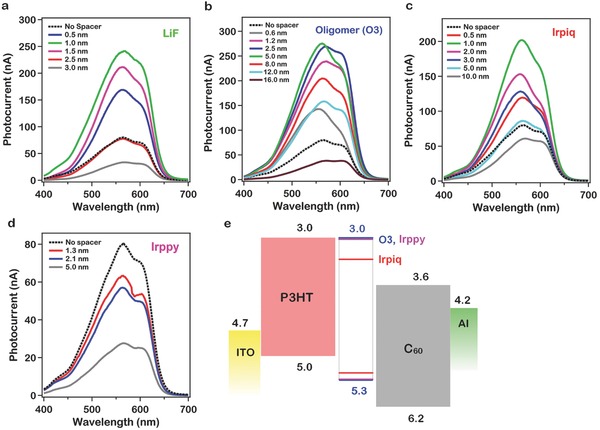
a–d) Photocurrent spectra measured for different thicknesses of LiF, O3, Irpiq, and Irppy. e) Energy levels for the spacer layers with respect to P3HT and C_60_.

**Figure** **3** describes the variation in the measured peak short‐circuit photocurrent (*λ* = 565 nm) as a function of spacer layer thickness for LiF, O3, Irpiq, and Irppy layers, normalized to the current for the structure without a spacer layer and the corresponding device model calculations. For each of the spacer layers used at the interface, we observed a different decay rate of the photocurrent with increasing thickness and encounter a unique mechanism that alters the interface recombination rate leading to either an increase (LiF, O3, and Irpiq) or decrease (Irppy) in the photocurrent.

**Figure 3:**
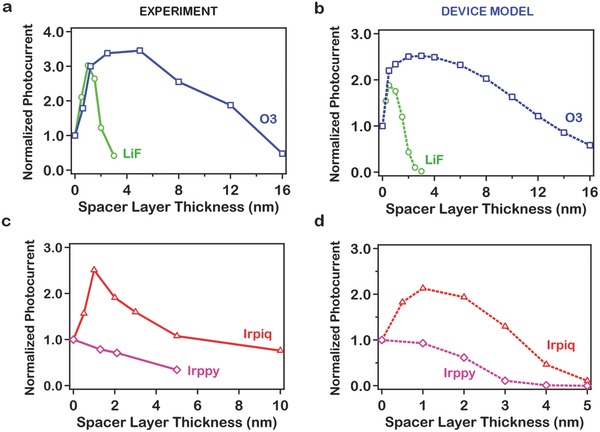
Peak photocurrent normalized to the photocurrent with no spacer layer as a function of spacer layer thickness from experiment and device modeling. a) Experimentally observed thickness dependence of the normalized photocurrent for LiF and O3 inserted devices. b) Model calculations for the thickness‐dependent photocurrent for LiF and O3 devices. c) Experimentally observe thickness dependence of the normalized photocurrent for Irpiq and Irppy and d) corresponding device model calculations for Irpiq and Irppy devices.

For LiF as a spacer layer (Figure 3a, green curve), with increasing thickness, we observed a sharp increase for ≈1 nm LiF thickness, which was followed by an exponential decrease in the photocurrent. We explain this using a simple tunneling mechanism that leads to the suppression of the CT state recombination, which decreases exponentially with increasing spacer layer thickness. Without a spacer layer, the exciton dissociation rate is orders of magnitude faster than other transition rates^6^, ^12^ and most importantly, the CT state recombination rate is comparable to, or faster than, the charge separation rate, and it is the dominant loss mechanism. Upon addition of a LiF layer, exciton dissociation occurs via tunneling across the LiF barrier. Increasing the thickness of the LiF layer lowers the exciton dissociation rate but since this rate is orders of magnitude faster than the exciton recombination rate, for sufficiently thin LiF layers (0–1 nm) exciton dissociation still dominates over exciton recombination. The LiF barrier reduces the CT state recombination rate so that charge separation dominates CT state recombination resulting in a large increase in the photocurrent. As the thickness of the LiF spacer layer is increased beyond ≈1.0 nm, the exciton dissociation rate is reduced below the exciton recombination rate, and the photocurrent decreases exponentially.

In sharp contrast to LiF devices, the measured photocurrent increases for devices with O3 spacer layers (Figure 3a, blue curve) with increasing O3 thickness up to a thickness of 5.0 nm and then decays at a rate much slower than that of LiF devices. We suggest that the exciton dissociation occurs via a two‐step process: (a) fast energy transfer of the exciton from donor across O3 to the acceptor and (b) back cascading of the hole to the donor. The hole has a favorable energy alignment for transfer from C_60_ to the P3HT and can cascade back to the P3HT via the O3. The presence of O3 works to spatially separate the electron and hole across the donor–acceptor interface, thereby strongly suppressing the CT state recombination. This manifests itself in a ≈350% increase in the photocurrent. Our photocurrent measurements support the recent ultrafast studies on donor–acceptor blends showing that exciton dissociation occurs via an excitation energy transfer mechanism where the exciton energy is resonantly transferred to the C_60_ followed by rapid hole transport from the C_60_ to the P3HT.^13^ The thickness dependence for O3 as the spacer layer is well described by using a 1/*L* ^6^ law, where, *L* is the thickness of the spacer layer (Figure 3b). The overall macroscopic charge transport, and thus the PCE, is also strongly correlated with the molecular level ordering and the morphology of the O3 (in‐plane or out‐of‐plane, transverse lamellar). The stacking determines the degree of spatial separation that the electron (on donor) and hole (on acceptor) experience after the energy transfer process has reached equilibrium. X‐ray diffraction (XRD) pattern for O3 shows (001), (002), (003) peaks indicating that O3 is well ordered and prefers a lamellar out‐of‐plane orientation with a *d*‐spacing of ≈4 nm. This facilitates charge separation of the electron and hole leading to enhanced PCE. (See Figure S2B, Supporting Information, for X‐ray diffraction patterns. A comprehensive analysis of the role of ordering at the interface will be described in another paper.)

The spacer layer thickness dependence of the photocurrent for Irpiq (Figure 3c, red curve) is quite different from that observed for both LiF and O3. Based on the energy level alignment in Figure 3c, we suggest that exciton dissociation occurs via energy cascading of the electron from P3HT to C_60_ assisted by Irpiq. The HOMO level for Irpiq lies in between the HOMO levels of P3HT and C_60_ allowing for a favorable transfer of the electron from P3HT to Irpiq and then to C_60_, but presents a barrier for the hole to be transferred to the C_60_ leading to a rapid separation of the electron and hole resulting in an increase in the photocurrent. The energy cascade can be further facilitated by the formation of long‐lived triplet state^9^ (Sections 8 and 10, Supporting Information, for discussion). Irppy molecule (Figure 3c, pink curve) is similar to Irpiq except for a different energy alignment such that the HOMO level is essentially degenerate with the HOMO level for P3HT, which does not facilitate the energy cascade of the electron from P3HT to C_60_ via Irppy. Thus, the photocurrent with the Irppy spacer drops in comparison to the photocurrent with no spacer layer and it decreases monotonically with increasing Irppy layer thickness. The thickness‐dependent photocurrent profiles for all the spacer layers are in excellent agreement with device modeling simulations (Figure 3b,d).

**Figure** **4**a,b illustrates the increase in the light *J*–*V* curves and extracted PCE under AM 1.5 illumination measured for spacer layer thickness and the corresponding PCE change as a function of spacer layer thickness. Note that the integrated photocurrent spectra are in excellent agreement with the measured *J*_SC_ from the current–voltage curves (see Figure S6 and Table S2, Supporting Information). We observed an increase in the *J*_SC_ and *V*_OC_ with the spacer layers suggesting that the presence of the spacer layers acts to suppress the CT state recombination. The increases in the *J*_SC_ and *V*_OC_ translate to a dramatic increase in the PCE by 2–5 times in comparison to devices without a spacer layer (Figure 4b). These increases in the PCE were achieved without any optimization of the bilayer device and reflect the lower end for efficiencies that are obtainable with bilayer devices.

**Figure 4:**
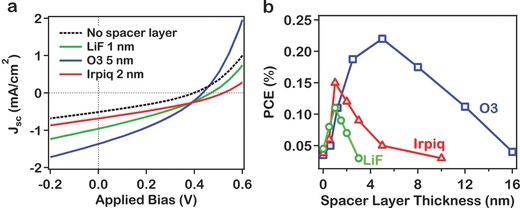
Increase in power conversion efficiency under AM1.5 standard solar radiation with functional spacer layers for our model bilayer device fabricated from P3HT (donor) and C_60_ (acceptor). a) Current density–voltage (*J*–*V*) curves characteristics measured under AM 1.5 illumination measured for devices with (solid curves) and without (dotted) interface spacer layers, b) overall power conversion efficiency (PCE) as a function of spacer layers thickness establishing the proof of principle that interface modification can suppress CT state recombination.

In order to validate these strategies beyond their demonstration in a model bilayer OPV device, we applied them to practically viable bulk heterojunction architectures using three different combinations of donor–acceptor material systems. **Figure** **5**a–d illustrates the BHJ device architecture, the corresponding energy level alignment, the current density–voltage curves under AM 1.5 illumination and the overall PCE as a function of loading weight percentage of the spacer layers, respectively (see Experimental Section for fabrication details). We observed that after addition of the spacer layers, there is a significant increase in the *J*_SC_, a slight increase in the *V*_OC_ for the O3 and Irpiq, and a corresponding decrease for Irppy (Figure 5c), consistent with our observations for model bilayer devices. The EQE spectrum is illustrated in Figure S11, Supporting Information, the integrated current density (11.2 mA cm^−2^) matches well with measured *J*_SC_. This corresponds to an increase in the average PCE from ≈4.1% (no spacer layer) to almost 6.9% (with interface spacer layers, max. 7.25%). Figure 5d illustrates how the average PCE varies over ten devices tested with the loading weight percentage of the spacer layers. The maximum PCE for the O3 spacer layer devices is obtained for 6 wt% loading and for 4 wt% for Irpiq, while for the Irppy doped device, the PCE monotonically decreases.

**Figure 5:**
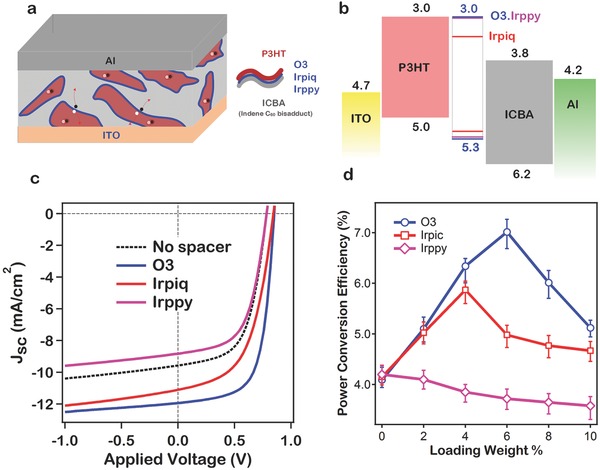
a) Bulk heterojunction architecture practically used for organic photovoltaic devices. P3HT (donor), spacer layers (O3, Irpiq, and Irppy) and indene–C_60_ bisadduct (ICBA, acceptor) were blended to form the active layer. b) Energy level alignment for the various components of the BHJ device. c) *J*–*V* characteristics of the OPV device with and without the presence of the various spacer layers under AM 1.5 illumination and d) power conversion efficiency as a function of loading weight% of the spacer layers.

In order to identify the role of the additive molecule (O3) in the BHJ solar cell, we performed thermal annealing (see Figure S13, Supporting Information) and impedance spectroscopy (Figure S14, Supporting Information). Based on our experimental observation, we suggest that O3 plays an important role in promoting efficient charge separation, which leads to the observed increase in the PCE (see Section 14, Supporting Information, for details). While the exact location of the additive O3 was difficult to determine through imaging techniques such as atomic force microscopy (AFM) and high resolution transmission electron microscopy (HRTEM), X‐ray diffraction (XRD) analysis of the BHJ device detects no observable change before and after adding the molecules. (See Figure S15, Supporting Information.) Similar results were also obtained for two other donor–acceptor systems: (i) P3HT (donor) and [6,6]‐phenyl‐C61‐butyric acid methyl ester (PC_61_BM, acceptor) and (ii) Poly[[9‐(1‐octylnonyl)‐9H‐carbazole‐2,7‐diyl]‐2,5‐thiophenediyl‐2,1,3‐benzothiadiazole‐4,7‐diyl‐2,5‐thiophenediyl] (PCDTBT):PC_71_BM are described in Figures S11 and S12, Supporting Information. The increase in performance by incorporating the interface spacer layers in commercially relevant BHJ architectures could possibly be due to the microscopic alignment of the O3 molecule similar to that observed in bilayer devices with O3 as spacer layer. These experiments demonstrate that the interface design strategies outlined here indeed lead to a dramatic increase in the overall PCE independent of the device architecture or the donor–acceptor polymer system.

In summary, we have discussed general interface design principles that allow for the control of fundamental rates of the critical interface electronic processes. This has been a long‐standing scientific bottleneck in the field of organic electronic devices. Using three distinct types of interface functional spacer layers between the active layers of an OPV device, we demonstrated that the PCE can be dramatically improved by as much as ≈2–5 times in a model bilayer device. These design strategies can be successfully extended to practical OPV architectures and result in an increase in the overall PCE from ≈4.0% to greater than 7.0%. The four functional interface spacer layer systems, LiF, O3, Irpiq, and Irppy reveal distinct trends in experiment, which indicates that the physical mechanisms of the interfacial processes that are being manipulated is unique for each of the spacer layers. From a broader photovoltaics community perspective, similar interface design strategies could be applicable to a wide range of hybrid material systems with organic–inorganic and inorganic–inorganic interfaces such as perovskite solar cells, atomic layered 2D interfaces, nanostructured systems such as quantum dots, single‐wall carbon nanotubes for the development of next generation, high efficiency light to energy conversion optoelectronic devices.

## Experimental Section

*Bilayer Device Fabrication*: Prepatterned indium tin oxide (ITO) glass slides were cleaned following a standard procedure for organic solar cell fabrication (sonication bath in DI water, acetone, and isopropyl alcohol for 15 min, respectively). After drying on a hotplate in air at 120 °C for 1 h the slides were cleaned with oxygen plasma for 3 min. A stock solution of PEDOT:PSS (Clevios H 4083) was spun cast on clean ITO slides at 6000 rpm for 40 s with a thickness of ≈40 nm as hole extraction/exciton blocking layer. PEDOT film was subjected to drying on a hotplate at 120 °C for 30 min and the slides were transferred to an Argon filled glovebox for active layer fabrication and cathode deposition. rr‐P3HT is dissolved in chlorobenzene at 4 mg mL^−1^ by stirring at room temperature inside the Ar fill glovebox for at least 12 h before using. The solution was spin coated on substrate at 1400 rpm for 35 s to form a 12 nm absorbing layer. (Layer thickness obtained by ellipsometry.) After the film has completely dried, the whole slide was transferred into vacuum chamber in the glovebox and pumped down to 2 × 10^−7^ Torr for interface layer (LiF, O3, or metal organic complex) and fullerene (C_60_ ≈ 35 nm) deposition by thermal evaporation. Note the interface layer and C_60_ has to be deposited in one run. After C_60_ deposition, the devices were taken out and placed on a shadow mask for cathode deposition (LiF ≈ 1 nm, Al ≈ 100 nm) in the same chamber. The active area was determined to be 0.03 cm^2^. All the film thickness (P3HT, C_60_, LiF, O3, and Ir(piq)_2_(acac), Ir(ppy)_3_ were determined by ellipsometry.

*Bulk‐Heterojunction Device Fabrication*: The bulk‐heterojunction solar cell was fabricated using the same protocol as described above in argon filled glovebox. The predissolved P3HT:ICBA (1:1) in *o*‐dichlorobenzene with P3HT concentration of 25 mg mL^−1^ was used as active layer formation. The spacer molecules were predissolved in chlorobenzene at 1 mg mL^−1^. Once the polymer/fullerene solution was fully dissolved after 10 h stirring at room temperature, the 100 μL in‐stock solution was mixed with 2, 4, 8, and 10 μL of the spacer molecule solution. And 10, 8, 6, and 2 μL solvent were added afterward to achieve the same polymer concentration for all solutions. The solution was further stirred for 1 h and was spin coated on top of PEDOT coated ITO slides at 1000 rpm for 30 s, allowed for drying in a glass petri dish for 10 min. Then whole device was then transferred to vacuum chamber pumped down to 2 × 10^−7^ Torr for cathode deposition by thermal evaporation. The whole device was postannealed at 150 °C for 10 min in the argon glovebox for crystal formation.

*Power Conversion Efficiency Measurement*: All solar cells were measured inside Ar filled chamber that was pumped down to 1 × 10^−6^ Torr. The shadow mask confined the device area of around 0.03 cm^2^ for cathode deposition. The same mask is used during device measurement to avoid edge effect for small area solar cell. Current–voltage sweeps were done using Keithley 2100 unit under simulated air mass 1.5 irradiation (100 mW cm^−2^) using a xenon‐lamp‐based solar simulator (Oriel LCS‐100). A NIST calibrated monocrystalline silicon solar cell (Newport 532, ISO1599) was used for light intensity calibration every time before measurement.

*Photocurrent Measurement*: The photocurrent was measured with a NIST calibrated monochromator (Acton SP2300) in AC mode. The light intensity was calibrated with a NIST calibrated photodiode (91005) as a reference each time before measurement. The monochromator was chopped at a frequency of 151 Hz. The photocurrent was collected through an preamplifier and a lock‐in amplifier as a function of incident wavelength.

## Supporting information

As a service to our authors and readers, this journal provides supporting information supplied by the authors. Such materials are peer reviewed and may be re‐organized for online delivery, but are not copy‐edited or typeset. Technical support issues arising from supporting information (other than missing files) should be addressed to the authors.

Supplementary
