# Supplementary material for: Interface Design Principles for High‐Performance Organic Semiconductor Devices
Source: Adv Sci (Weinh). 2015 Mar 23;2(6):1500024. doi: 10.1002/advs.201500024 (PMC5115400; doi:10.1002/advs.201500024)
Supplement: Supplementary file 1 — Supplementary [file ADVS-2-0i-s001.pdf]

Copyright WILEY-VCH Verlag GmbH & Co. KGaA, 69469 Weinheim, Germany, 2015.

ADVANCED SCIENCE
Open Access

Supporting Information

for *Adv. Sci.*, DOI: 10.1002/advs.201500024

Interface Design Principles for High-Performance Organic Semiconductor Devices

Wanyi Nie, Gautam Gupta, Brian K. Crone, Feilong Liu, Darryl L. Smith, P. Paul Ruden, Cheng-Yu Kuo, Hsinhan Tsai, Hsing-Lin Wang, Hao Li, Sergei Tretiak, and Aditya D. Mohite**

Supplementary Materials for

Interface Design Principles for High Efficiency Organic Semiconductor Devices

Wanyi Nie*, Gautam Gupta, Brian K. Crone, Feilong Liu, Darryl L. Smith, P. Paul Ruden, Cheng-Yu Kuo, Hsinhan Tsai, Hsing-Lin Wang, Hao Li, Sergei Tretiak and Aditya D. Mohite*

*Correspondence to: Aditya D. Mohite (amohite@lanl.gov) and Wanyi Nie (wanyi@lanl.gov)

1 Absorption UV-Vis

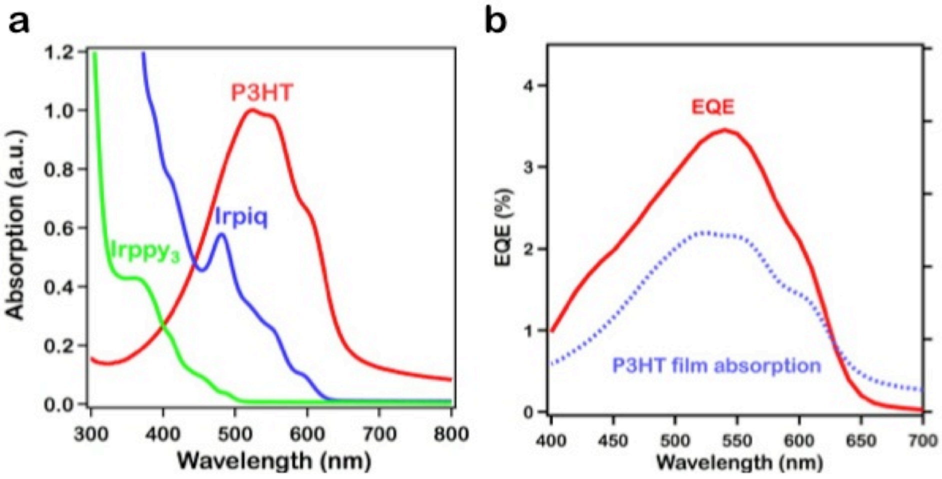

Figure S1 a, Normalized UV-Vis absorption spectrum for solution of O3, Ir(ppy)$_3$, Ir(piq)$_2$(acac) and rr-P3HT in chlorobenzene **b,** P3HT film absorption and External quantum efficiency for P3HT/C_{60} device

The UV-Vis absorption has been measured for P3HT, Irpiq and Irppy solution in chlorobenzene are shown in Fig. S1a. Fig.S1 b shows the external quantum efficiency of one standard bilayer device with P3HT (10 nm)/C_{60} (35 nm). The EQE spectrum is consistent with the P3HT film

absorption shown in dotted line in Fig.S1b. The absorption spectrum and EQE is consistent with literature report by other groups.

2 Characterization on thermal deposited Oligothiophene (O3) films

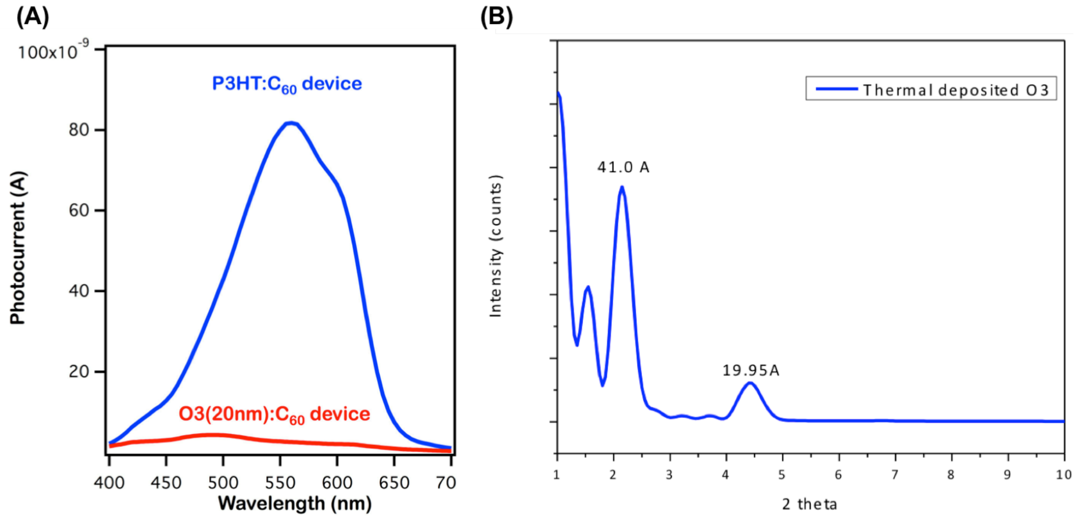

Figure: S2 (A) Photocurrent for (red) O3/C_{60} bilayer device in a device structure of: ITO/PEDOT: PSS/O3(20 nm)/C_{60}(35 nm)/LiF/Al and (blue) P3HT/C_{60} bilayer device in the structure of: ITO/PEDOT:PSS/P3HT(12 nm)/C_{60}(35 nm)/LiF/Al (B) X-ray diffraction on (XRD) O3 film deposited by thermal evaporation

The photocurrent measured from a bilayer device composed with O3 (20 nm)/C_{60} (35 nm) shown in Fig. S2 (red curve). Compared to the standard P3HT/C_{60} device (blue curve), the O3 device shows 20 times lower in peak photocurrent, which suggests that for P3HT/O3/C_{60} device, the majority photocurrent contribution is comes from P3HT that has 20 times higher absorption than O3 film.

3 Cyclic voltammetry measurement on Oligothiophene (O3) for the determination of HOMO-LUMO level

The LUMO level of Oligothiophene (O3) was determined using a combination of cyclic voltammetry (CV) and linear absorption spectroscopy(*1, 2*). O3 was dissolved in anhydrous acetonitrile and 0.1 M tetrabutylammonium perchlorate (TBAP) was used as an electrolyte. Three-electrode configuration was used with Pt as working electrode, Ag/$AgNO_3$ as reference electrode and Pt Gauze as counter electrode. Cyclic voltagram was acquired at a scan rate of 100 mV/s. Redox values for Ferrocene were obtained and the oxidation potential ($\mathbf{E_{ferrocene}}$) was used as calibration standard. The $\mathbf{E_{ox}}$ potential was measured by the intersection of the extrapolated tangent lines of the redox curve illustrated in Fig. S3.

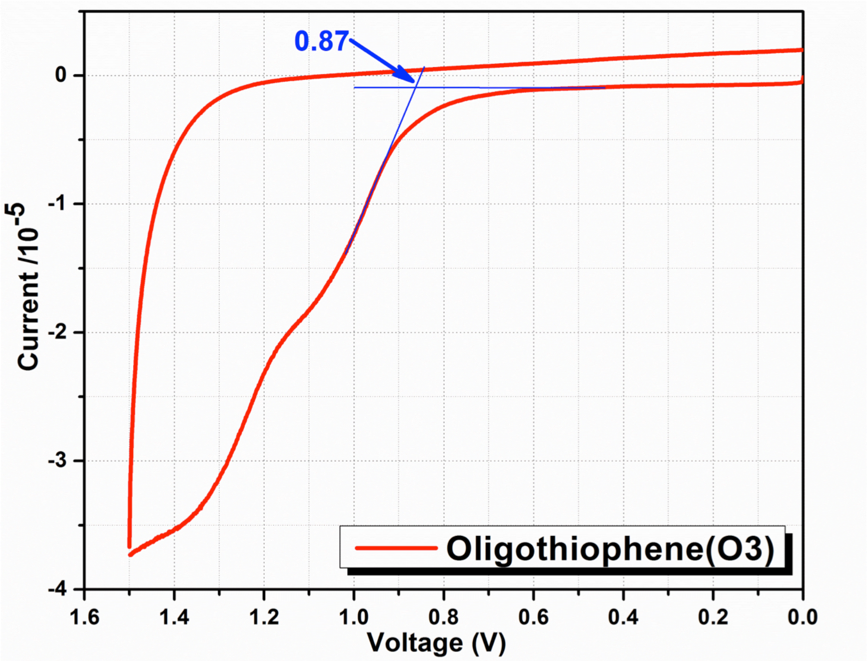

Figure: S3 Cyclic voltammetry measurement on Oligothiophene (O3).

The HOMO level is given by equation (1).

HOMO= -(E_{oxd} - $E_{ferrocene}$ + 4.8) …………………………(1)

Band-gap energy was estimated by extrapolating tangent lines at the absorption edge of the O3 absorption spectra and using equation (2). The LUMO level for O3 was determined using equation (3).

$$E_g = 1240/\lambda_{onset} \quad \text{……………………………………..} \ (2)$$

$$\text{LUMO} = \text{HOMO} + E_g \quad \text{……………………………..} \ (3)$$

Table S1 summarizes the HOMO and LUMO energy levels for O3 and the different parameters used to determine them.

Table: S1 HOMO and LUMO Energy Level and Band Gap Determined from UV-Vis absorption spectra and cyclic voltammetry measurements on O3.

Oligomer	E_{oxd} (V)[a]	E_{red} (V)	λ_{onset} (nm)[b]	E_g (eV)[c]	HOMO (eV)	LUMO (eV)
**O3**	+0.87	not available	550	2.25	-5.27	-3.02

[a] First oxidation potential value from cyclic voltammetry measurement.

[b] Onset point or band-edge of the UV-Vis absorption spectra of the O3 film.

[c] Energy gap (E_g) measured according to the band-edge of UV-Vis absorption spectra of the deposited thin film(E_g =1240/λ_{onset} eV).

4 Mtdata/Bphen bilayer device dark IV curve and photocurrent

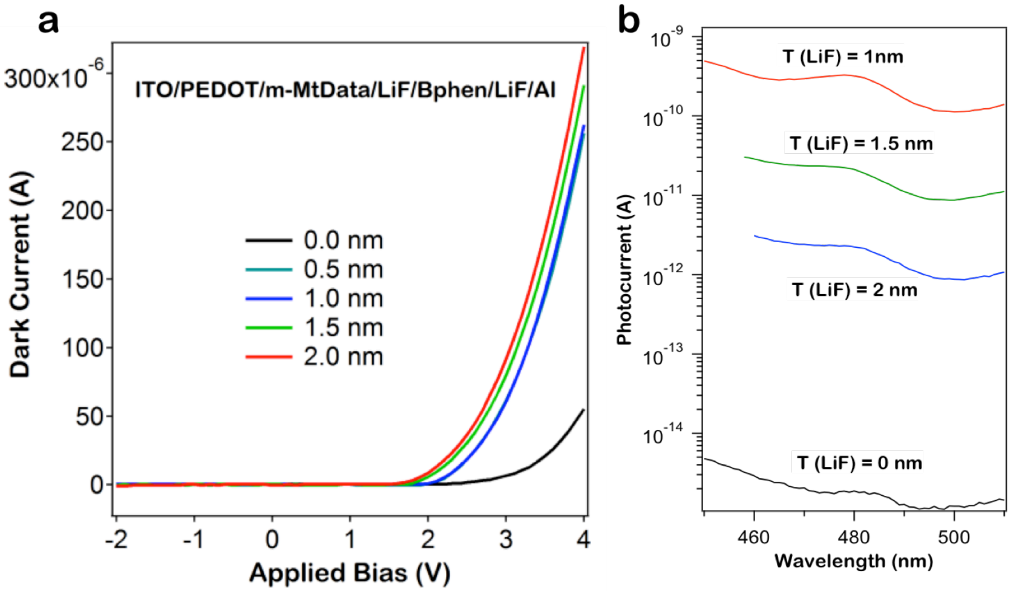

Figure: S4 a, dark IV curve and **b,** short-circuit photocurrent for bilayer device composed with m-Mtdata (50 nm) /LiF(x nm)/Bphen (50 nm) , x=0 nm, 0.5 nm, 1 nm, 1.5 nm, 2 nm

As described in the main text, the interface modification strategy has been applied to several organic donor-acceptor pairs. Fig. S4 gives an example of them. The bilayer device is comprised with m-Mtdata/Bphen donor-acceptor pair, the interface was modified with LiF for 0~2 nm thickness. The dark current-voltage curve is shown in Fig. S4a device with and without LiF. The results showing with LiF the dark current-voltage curve increase under forward bias, which is consistent observation with the P3HT/C_{60} device with LiF as spacer. In Fig. S4b, the

photocurrent shows 5 orders of magnitude of improvement by adding 1 nm LiF, which suggests the CT state recombination can be suppressed in this system as well.

5 Optical absorption of bilayer device without and with LiF as spacer.

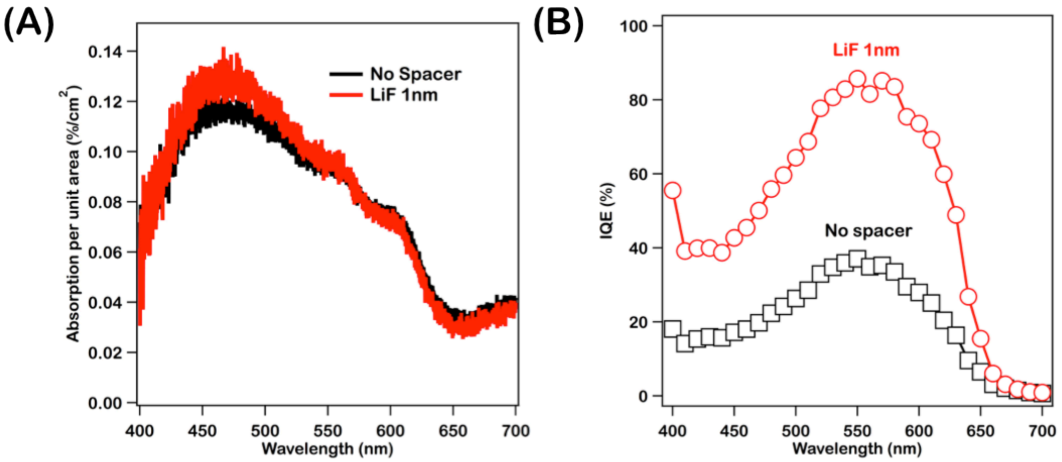

Figure S5: (A) and (B) shows the absolute absorption and IQE measured using an integrating sphere for devices with and without a spacer layer respectively.

Furthermore, we measured the absolute absorption and the internal quantum efficiency (IQE) for our devices. We followed a standard procedure for the absolute absorption measurement by Burkhard et al. (*3*) Briefly, we took an integration sphere coupled to a CCD camera, and used a Tungsten white-light source for illumination. The white light source was focused into the integration sphere and we measured the spectrum without any sample as reference. Then we measure the spectrum with the sample (ITO/PEDOT/P3HT:C_{60} without or with LiF/O3 spacer) in the center of the sphere (the sample is kept in a small angle with the incident light). The absorption is the total transmitted and reflected light that was collected by the integration sphere over the total incident light (without sample). Fig. S5 A shows the measured absorption spectrum for the no spacer device and for the one with a1nm LiF spacer layer. The presence of the spacer layer does not change the absorption. Normalizing our EQE data by the absorption we get IQE

(see Fig. S5 B). The device with the LiF spacer layer shows an IQE 2.5 times higher than the no spacer layer device. This clearly suggests suppression of interface recombination by adding LiF is the underlying process that gives the large enhancement and is not due to any changes in the optical density of the film.

6 IV curves under AM 1.5 illumination and Integrated Jsc from EQE

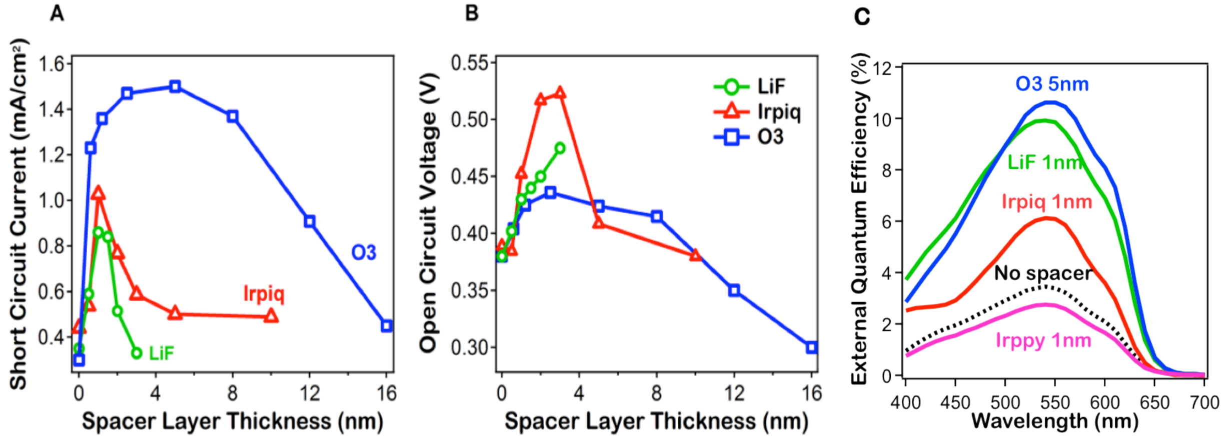

Figure: S6 A, The short circuit current density (J_{SC}) and **B,** open circuit voltage (V_{OC}) extracted from light J-V curve under A.M. 1.5 solar illumination for bilayer devices with spacer layer at various thicknesses. **C** shows the external quantum efficiency for these devices measured under short-circuit conditions. Integrating the EQE yields the short-circuit current density measured in **A** for the specific layer thickness.

Table S2 : Solar cell parameters extracted from IV curves under AM1.5 illumination for bilayer device with no spacer, 1nm LiF, 5nm O3, 1nm Irpiq and 1nm Irppy. The Jsc (Cal. From EQE) is the Jsc value integrated from EQE over the spectrum under short circuit condition.

Spacer layer	Voc (V)	Jsc (mA/cm^2) AM 1.5	Jsc (cal. from EQE) (mA/cm^2)	Fill Factor (%)	PCE (%)
**No spacer**	0.374	0.48	0.44	30.6	0.05
**LiF 1nm**	0.42	1.14	1.06	30.5	0.12
**O3 5nm**	0.41	1.4	1.36	30.7	0.15
**Irpiq 1nm**	0.43	0.70	0.78	27.6	0.10
**Irppy 1nm**	0.375	0.34	0.32	30.15	0.048

7 Device modeling

We use a previously developed device model (*4, 5*) to interpret the current-voltage measurements and predict trends arising from the complex competition of the underlying interface charge transport and recombination rates. Considering for example a bilayer P3HT/C_{60} device, under forward bias in the dark, electrons are injected into the C_{60} layer and holes are injected into the P3HT layer. The electrons and holes are transported to the donor-acceptor interface where they form CT states, which subsequently recombine (see Fig. S7a). The current flow is limited by the CT state formation rate from electrons in C_{60} and holes in P3HT and the CT state recombination rate. Thus, comparing dark current-voltage characteristics between devices with and without a spacer layer indicates how the interface layer influences charge transfer and recombination rates. Under AM 1.5 illumination there is photocurrent (see Fig. S7b). Excitons in P3HT diffuse to the interface where they convert to the CT state.

The CT state can either dissociate into an electron in C_{60} and a hole in the P3HT yielding a photocurrent, or it can recombine yielding no current. The CT state dissociation rate is related to the CT state generation rate from electrons in C_{60} and holes in P3HT by detailed balance. Except for comparatively thick donor/accptor layers, exciton diffusion to the interface and subsequent dissociation to form the CT state are efficient and photocurrent is limited by a competition between CT state recombination and dissociation. Comparison of current-voltage characteristics of devices with and without the addition of an interface spacer layer shows how transition rates for CT state recombination and CT state dissociation can be altered to achieve efficient photocurrent generation.

Fig. S7c-d compares the measured dark and illuminated current-voltage characteristics for devices with: *no spacer layer, 1 nm LiF layer and 5 nm O3 layer*. For these structures, exciton diffusion and dissociation to form the CT state is efficient and photocurrent is limited by

the competition between CT state dissociation and recombination. We observe experimentally that the dark current is increased by addition of both the LiF and O3 layers. This result suggests that the spacer layer increases the CT state formation rate from electrons in C_{60} and holes in P3HT. The illuminated current-voltage results show that the incorporation of both the LiF and O3 spacer layers also dramatically improves the photocurrent. This indicates that both the LiF and O3 spacer layers increase the ratio of CT state dissociation to CT state recombination and that this increase is greater for O3.

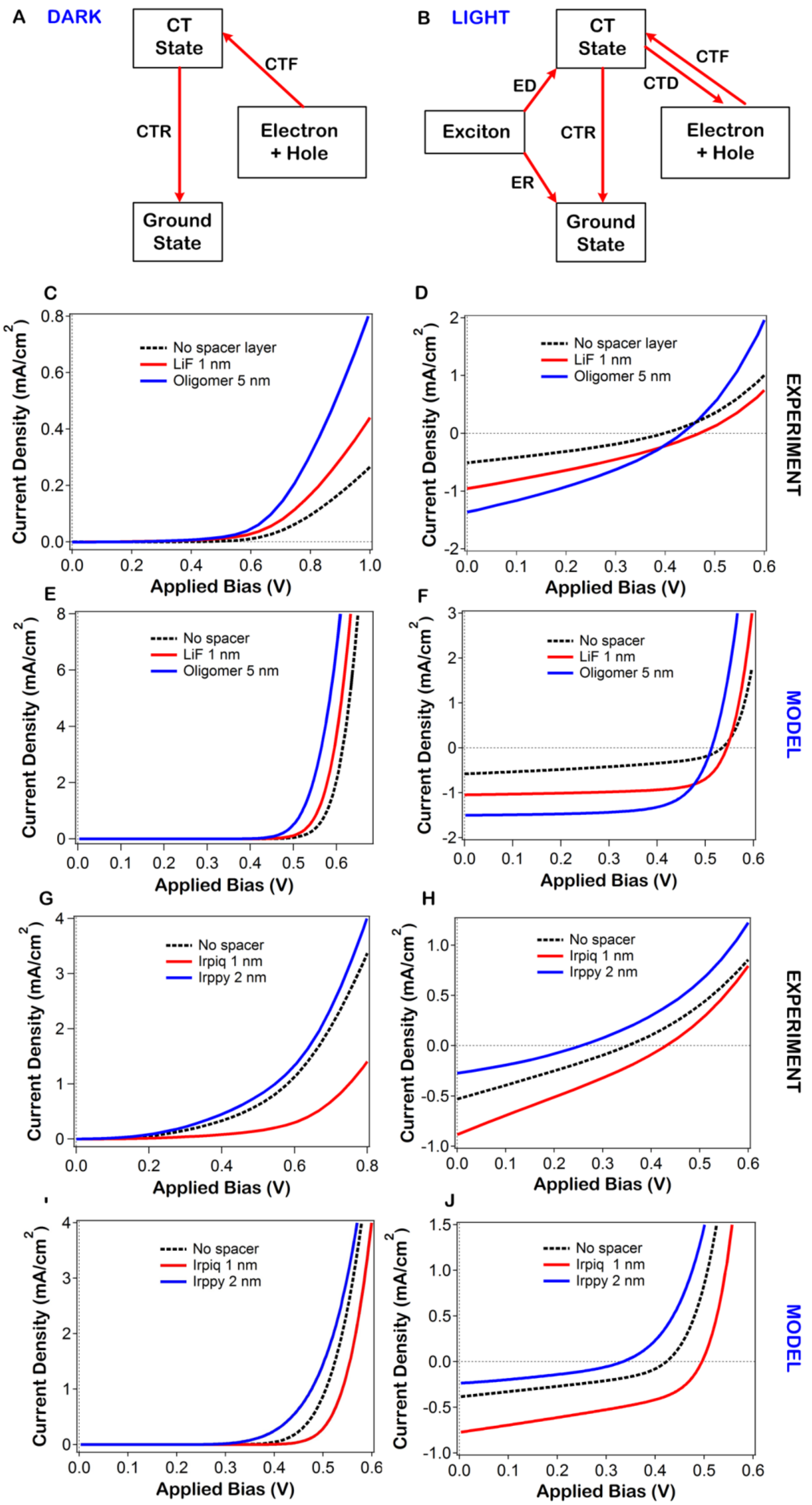
A DARK
CT State
CTF
CTR
Electron + Hole
Ground State
B LIGHT
CT State
ED
CTF
CTD
Exciton
CTR
Electron + Hole
ER
Ground State
C
No spacer layer
LiF 1 nm
Oligomer 5 nm
Current Density (mA/cm2)
Applied Bias (V)
D
No spacer layer
LiF 1 nm
Oligomer 5 nm
Current Density (mA/cm2)
Applied Bias (V)
EXPERIMENT
E
No spacer
LiF 1 nm
Oligomer 5 nm
Current Density (mA/cm2)
Applied Bias (V)
F
No spacer
LiF 1 nm
Oligomer 5 nm
Current Density (mA/cm2)
Applied Bias (V)
MODEL
G
No spacer
Irpiq 1 nm
Irppy 2 nm
Current Density (mA/cm2)
Applied Bias (V)
H
No spacer
Irpiq 1 nm
Irppy 2 nm
Current Density (mA/cm2)
Applied Bias (V)
EXPERIMENT
J
No spacer
Irpiq 1 nm
Irppy 2 nm
Current Density (mA/cm2)
Applied Bias (V)
No spacer
Irpiq 1 nm
Irppy 2 nm
Current Density (mA/cm2)
Applied Bias (V)
MODEL

Figure: S7 Device model describing the predicted current-voltage characteristics with and without interface spacer layers. (A) and (B) Schematics of interfacial processes in dark and under illumination, respectively. **(C) and (G)** Current-voltage curves measured in the dark. **(D) and (H)** Current-voltage curves measured under illumination. **(E) and (F)** Calculated current-voltage curves in the dark. (I) and (J) Calculated current-voltage curves under illumination. **(C)-(F)** LiF and O3. **(G)-(J)** Irpiq and Irppy.

Fig. S7 E-F show calculated current-voltage curves in the dark and under illumination respectively for the three cases with transition rate coefficients adjusted to the experimental results. The transition rates with the interface processes; CT state recombination, CT state dissociation and CT state formation is also observed to be field dependent. As the applied forward bias increases, the change in the local electric field at the interface suppresses CT state dissociation and enhances CT state recombination and CT state formation rates. For simplicity we assume linear field dependence for the corresponding kinetic coefficients in the device model. The increase in the CT state formation rate by the interface layers is specific to this donor-acceptor system. For example, in the Tetracene/C_{60} system (*6*) the dark current was found to decrease with incorporation of a LiF spacer layer. For the P3HT/C_{60} system, the LiF or O3 interface layer may improve the interface smoothness and facilitate the in-plane carrier transport required for CT state formation. Under large forward bias, the calculated currents increase more rapidly than the experimental ones. This is probably due to series resistance effects associated with contacts, which are not included in the device model.

Fig. S7 G-H compares measured dark and illuminated (AM 1.5) current-voltage characteristics for devices with: *no spacer layer, 1 nm Irpiq layer and 2 nm Irppy layer*. The measured dark current is increased with the addition of the Irppy layer but decreased with the Irpiq layer. This result shows that the CT state formation rate is increased by the Irppy layer but decreased by the Irpiq layer. The illuminated current-voltage results show that the incorporation of the Irpiq layer improves the photocurrent but incorporation of the Irppy layer reduces the

photocurrent. Thus, the ratio of CT state dissociation to CT state recombination is increased by the Irpiq layer, but decreased by the Irppy layer. Fig. S7 I-J illustrates the respective current-voltage curves calculated in the dark and under illumination for the three cases with transition rates adjusted to the experimental results. The model results are in good qualitative agreement with the experimental current-voltage curves.

8 Theoretical simulations of ionization potential and electron affinity, singlet and triplet excitations

The ionization potential (IP) and electron affinity (EA) simulations have been calculated using density functional theory (DFT) for three thiolated oligothiophene derivatives (terthiophene, tetrathiophene, and pentathiophene), Fullerene, Iridium complexes [$Ir(piq)_2acac$ and $Ir(ppy)_3$], and poly(3-hexylthiophene-2,5-diyl) (P3HT) that contains 10 thiophenes. Ground state geometries of neutral molecules, anions and cations have been optimized using 6-31G* basis set and two hybrid functionals, widely used B3LYP and range-corrected ωB97X-D, which is known for its accuracy in large conjugated molecules with delocalized π-electrons. All simulations in this work have been carried out in solution of dichloromethane, whose dielectric constant ε_r=9.08 ε_0. Computations in solutions use self-consistent reaction field (SCRF) approach with solvation model of the conductor-like polarizable continuum model (C-PCM), which is known to be both accurate and efficient. All simulations have been conducted with Gaussian 09 package.

The ground state energies at optimized geometries of neutral molecules, cations and anions, in vacuum and in solutions, have been tabulated in Table S3. The ionization potential (IP) is calculated as $IP=E_{cation}-E_{neutral}$, whereas the electron affinity (EA) is calculated as $EA=E_{neutral}-E_{anion}$. The calculated IP&EA for molecules of interested are tabulated in Table S4.

B3LYP/6-31G*	neutral (a.u.)	cation (a.u.)	anion (a.u.)
P3HT	-7878.1891	-7878.0250	-7878.2777
O3	-2330.0276	-2329.8421	-2330.1004
O4	-2881.8441	-2881.6645	-2881.9249
O5	-3433.6607	-3433.4844	-3433.7464
Ir(piq)$_2$(acac)	-1714.7498	-1714.5615	-1714.8275
Ir(ppy)$_3$	-1658.9761	-1658.7933	-1659.0273
C60	-2286.1756	-2285.9621	-2286.2919

ωB97X-D/6-31G*	neutral (a.u.)	cation (a.u.)	anion (a.u.)
P3HT	-7876.9205	-7876.7258	-7876.9891
O3	-2329.7192	-2329.5234	-2329.7870
O4	-2881.4650	-2881.2725	-2881.5393
O5	-3433.2108	-3433.0195	-3433.2884
Ir(piq)$_2$(acac)	-1714.2222	-1714.0266	-1714.2981
Ir(ppy)$_3$	-1658.4782	-1658.2898	-1658.5126
C60	-2285.4570	-2285.2249	-2285.5683

Table S3. Ground-state energies of neutral molecules, cations and anions, computed in solution of dichloromethane using functional B3LYP (top) and ωB97X-D (bottom).

	Ionization potential (IP) (eV)		Electron affinity (EA) (eV)	
	**B3LYP**	**ωB97X-D**	**B3LYP**	**ωB97X-D**
P3HT	4.4677	5.2985	2.4098	1.8672
O3	5.0478	5.3273	1.9793	1.8451
O4	4.8890	5.2376	2.1972	2.0204
O5	4.7964	5.2052	2.3333	2.1116
Ir(piq)$_2$(acac)	5.1238	5.3236	2.1138	2.0636
Ir(ppy)$_3$	4.9725	5.1273	1.3927	0.9357
C60	5.8097	6.3156	3.1645	3.0296

Table S4. The ionization potential (IP) and the electron affinity (EA) of thiolated Oligothiophene, P3HT, fullerene, and Iridium complexes. Computations are performed in dichloromethane solution using functional B3LYP and ωB97X-D.

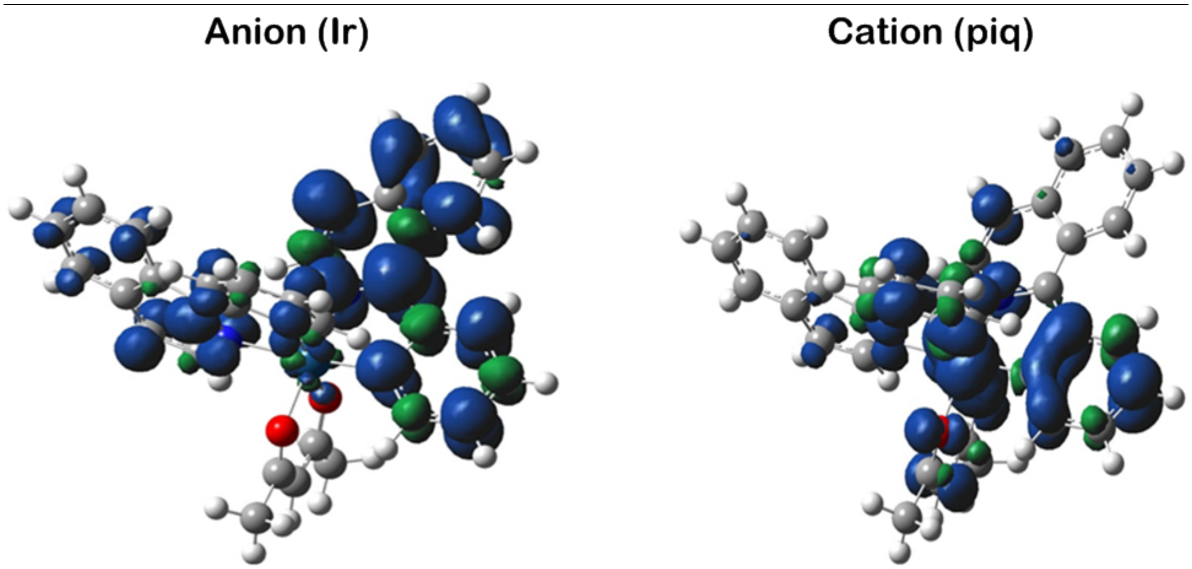

Figure S8 Spin density plots of anion (left) and cation (right) of Ir(piq)$_2$(acac) in dichloromethane solution simulated at quantum chemical level B3LYP/6-31G*.

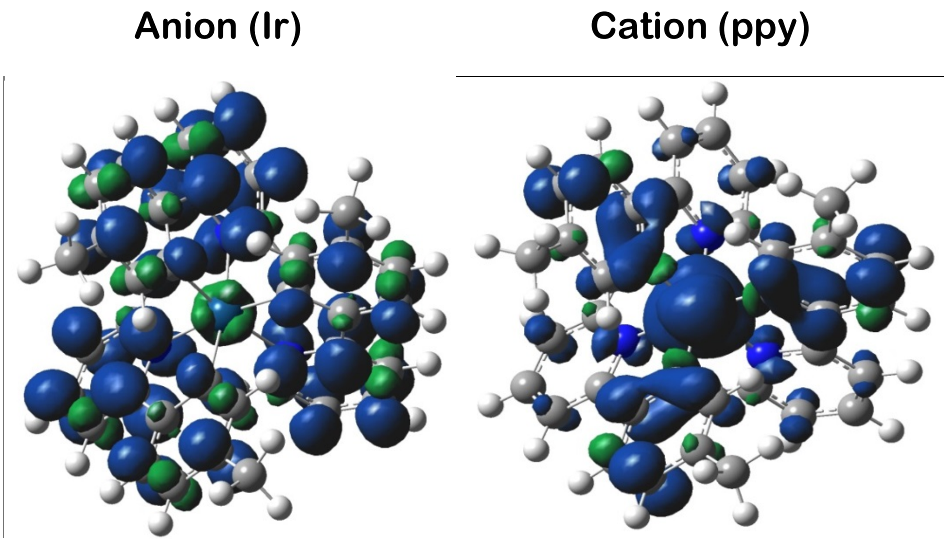

Figure S9 Spin density plots of anion (left) and cation (right) of Ir(ppy)$_3$ in dichloromethane solution simulated at quantum chemical level B3LYP/6-31G*.

In addition, the lowest then singlet and triplet electronic excitations of the two Iridium complexes of interest in dichloromethane solutions have been calculated using time-dependent density functional theory (TDDFT) at the same quantum-chemical levels.

	Ir(piq)$_2$(acac)		Ir(ppy)$_3$		fullerene	
State	Singlet	Triplet	Singlet	Triplet	Singlet	Triplet
1	2.5193 (0.03)	2.1091	3.0191 (0.02)	2.7012	2.0948	1.5939
2	2.6708 (0.07)	2.2514	3.0740 (0.01)	2.7121	2.0949	1.5940
3	2.7258 (0.04)	2.4716	3.0746 (0.01)	2.7123	2.0950	1.5940
4	2.8188 (0.03)	2.6146	3.2202 (0.05)	3.0237	2.0950	1.9240
5	3.0178 (0.08)	2.7926	3.2207 (0.05)	3.0491	2.1057	1.9240
6	3.2173 (0.07)	2.7985	3.2458 (0.01)	3.0501	2.1058	1.9240
7	3.4472 (0.02)	2.8796	3.3092 (0.06)	3.1438	2.1058	1.9505
8	3.4577 (0.03)	2.9099	3.3096 (0.06)	3.1441	2.1060	1.9505
9	3.5639 (0.13)	3.0744	3.3921 (0.01)	3.1694	2.1061	1.9506
10	3.5740 (0.02)	3.2992	3.5502 (0.09)	3.2963	2.1061	1.9506

Table S5. Excitation energies (in eV) of singlet and triplet excited states in the Iridium complexes and fullerene calculated at B3LYP/6-31G*. Oscillator strengths of singlet excitations are listed in the parenthesis. Oscillator strengths of electronic excitations in fullerene are vanishing.

	Ir(piq)$_2$(acac)		Ir(ppy)$_3$		fullerene	
State	Singlet	Triplet	Singlet	Triplet	Singlet	Triplet
1	3.1683 (0.07)	2.3263	3.7007 (0.12)	2.9385	2.6757	1.7653
2	3.3626 (0.17)	2.4227	3.7098 (0.07)	2.9390	2.6758	1.7655
3	3.5755 (0.07)	3.0068	3.7102 (0.07)	2.9413	2.6759	1.7655
4	3.7397 (0.02)	3.0383	4.0508 (0.12)	3.5767	2.7353	2.4535
5	3.7804 (0.05)	3.1875	4.0513 (0.12)	3.6099	2.7354	2.4535
6	4.0634 (0.05)	3.3606	4.1266 (0.03)	3.6102	2.7354	2.4536
7	4.0972 (0.23)	3.5305	4.1718 (0.01)	3.7700	2.7729	2.5841
8	4.1635 (0.22)	3.5761	4.1723 (0.01)	3.7703	2.7729	2.5842
9	4.2185 (0.05)	3.6149	4.2428 (0.05)	3.7811	2.7729	2.5842
10	4.3609 (0.02)	3.6984	4.4393 (0.05)	3.8810	2.7730	2.5843

Table S6. Excitation energies (in eV) of singlet and triplet excited states in the Iridium complexes and fullerene calculated at ωB97X-D/6-31G*. Oscillator strengths of singlet excitations are listed in the parenthesis. Oscillator strengths of electronic excitations in fullerene are vanishing.

9 Discussion about charge transfer mechanism for Oligothiophene (O3) and heavy atoms at D-A interface

We consider two mechanisms that could lead to the thickness dependence that we observe for the O3 spacer layers. First, we consider that the thickness dependence of the photocurrent is limited by the exciton diffusion length (L_d~15 nm). This in our case would result from the total thickness of the P3HT (~10 nm) and the O3 (5 nm). The excitons generated in the P3HT migrate through the O3 to reach the O3/C_{60} interface where they dissociate to form the CT state. Here we note that the absorption in O3 is much weaker than in P3HT, and is shown from photocurrent measurements on O3 in Fig. S2 of SI). Thus if the total thickness of the P3HT and O3 is greater than the exciton diffusion length, then a drop in the photocurrent signal would be expected consistent with the experimental observations. However, this mechanism requires that excitons can resonantly diffuse from the P3HT to the O3. The effective optical gap for the O3 is considerably larger than P3HT with negligible spectral overlap (See absorption spectra Fig. S1 in SI). Thus the photoexcited excitons would be required to gain additional energy in order to diffuse into the O3, which is not plausible.

10 Role of spin-orbit coupling and triplet states in using Metal organic as spacer layers

The energy cascade process can be further facilitated by the formation of long-lived triplet state. The primary single CT state with a hole on P3HT and an electron on Irpiq (or hole on Irpiq) and an electron on C_{60} can undergo rapid intersystem crossing to a long-lived triplet CT state due to a close proximity on the heavy atom. (*7*) Indeed, calculated spin-density plots (in Fig. S8 and Fig. S9) show that the hole is strongly localized on the Ir whereas the electron is essentially localized on the -piq ligands. Stabilization into long-lived triplet state further promotes cascade-like separation of the electron from the hole leading to an increase in the

overall photocurrent efficiency. As the thickness of the Irpiq is increased to 1 nm, the photocurrent reaches a peak value and then for and then for >1 nm thickness of Irpiq, begins to drop reaching a level close to that without a spacer layer. Such drastic reduction in the photocurrent efficiency in likely due to quenching of the exciton and reduced charge mobility in the Irpiq layer. Furthermore, the absorption spectrum and the electronic structure calculations of Irpiq (see Table S5 and S8 in SI) suggests a manifold of low-lying intra-molecular singlet states, where transfer of excitons from P3HT is energetically feasible and more probably with increase in the spacer layer thickness. However these intra-molecular singlet states are expected to undergo rapid intersystem crossing into low-lying intra-molecular triplet states acting as quenching sites. (*7*) Moreover, with increasing spacer layer thickness, an electron and hole in the Irpiq layer will undertake an increasing number of hopping steps to achieve complete spatial separation of carriers to C_{60} and P3HT regions respectively. (*8, 9*)Due to highly localized electron and hole states (see Table S5 and S8 in SI) such transport is not very efficient and can lead to a further reduction in the photocurrent with increasing thickness of Irpiq.

11 External quantum efficiency for bulk heterojunction device (P3HT:ICBA) with oligothiophene and Irpiq modification

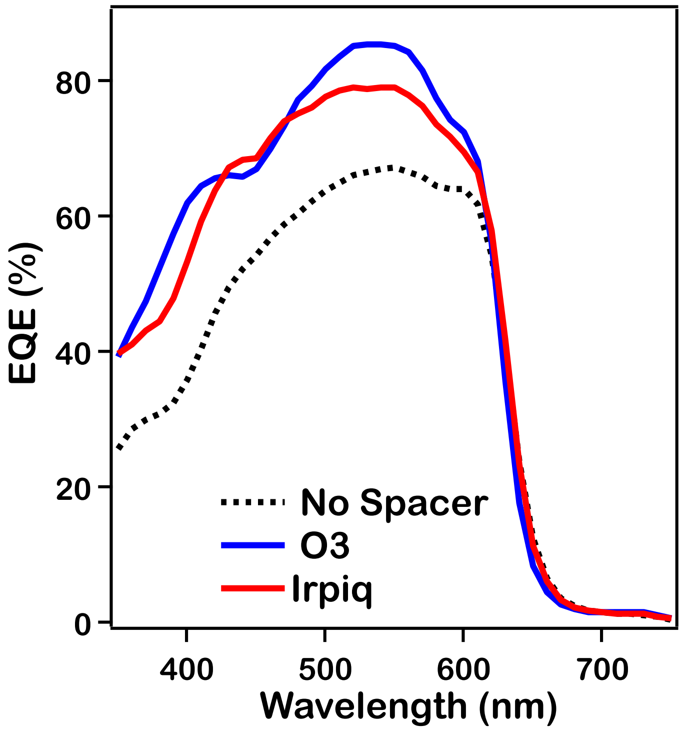

Fig. S10 External quantum efficiency (EQE) spectrum for bulk-heterojunction devices with P3HT: ICBA without and with spacer layer modification

External quantum efficiency is shown in Fig. S10 for BHJ devices without and with spacer molecules. By adding 6% O3, the EQE increases by 25%, which is consistent with the light current-voltage curve results. Similarly, the EQE has increased by 20% by adding 4% Irpiq that is consistent with the observation from light current-voltage curves. Note the integrated current density calculated from the EQE (J_{SC}=11.2mA/cm^2) spectrum matches with the J_{SC} measured out of the light current-voltage curves. The 6% difference measured from EQE with measured J_{SC} can be due to the spectrum mis-match from the monochromator and the AM 1.5 white light.

Table S7 Avergage solar cell parameters extracted from the light J-V curves in Fig.5 in the main text over 10 devices

Spacer layer	Avg. Voc (V)	Avg. Jsc (mA/cm^2) AM 1.5	Max. Jsc (cal. from EQE) (mA/cm^2)	Fill Factor (%)	Avg PCE (%)	Max. PCE (%)
**No spacer**	0.783±0.025	9.57±0.3	9.17	58.16±5.5	4.12	4.89
**O3**	0.859±0.02	11.4±0.5	11.25	65.5±4.2	6.89	7.21
**Irpiq**	0.844±0.013	11.0±0.22	10.89	60.1±6.3	5.57	6.24

12 Light IV curve and external quantum efficiency for bulk-heterojunction devices ($P3HT:PC_{61}BM$)

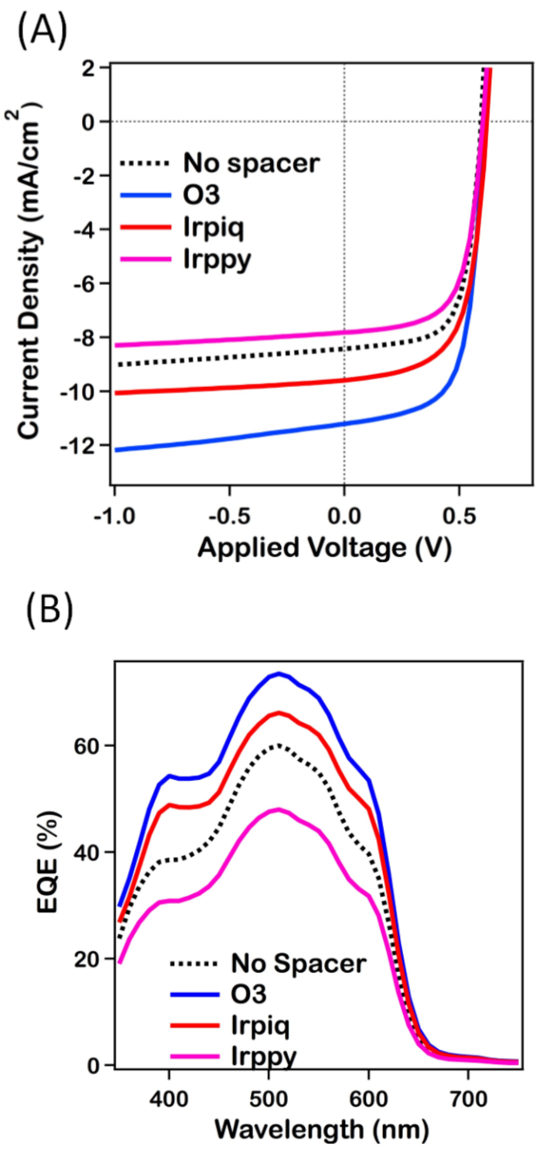

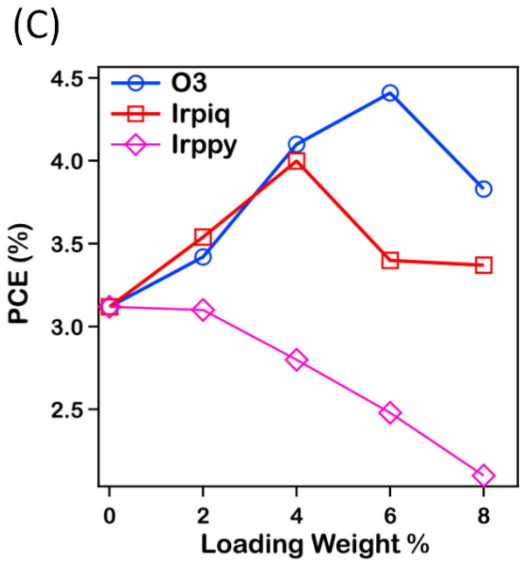

Fig. S11 (A) IV characteristics under AM 1.5 illumination and (B) external quantum efficiency for bulk-heterojunction solar cell with P3HT: PCBM as absorber without and with spacer layer modification; (C) Average power conversion efficiency as a function of spacer layer weight percentage

Similar bulk-heterojunction devices were tested with P3HT: PCBM system with spacer layers added. The light current-voltage characteristics and external quantum efficiency are shown in Fig. S11 (A) and (B). By adding 6% O3 or 4% Irpiq, the J_{SC} again showed improvement but Irppy decreased the performance. Fig. S11 (C) shows the statistical summarization of power conversion efficiency for BHJ devices with those three spacer materials. The PCE show same trend as in P3HT: ICBA devices. The PCE was increased by 20% using O3 as spacer layer, from ~3.5% up to ~5%.

13 Light IV curve and external quantum efficiency for bulk-heterojunction devices ($PCDTBT:PC_{71}BM$)

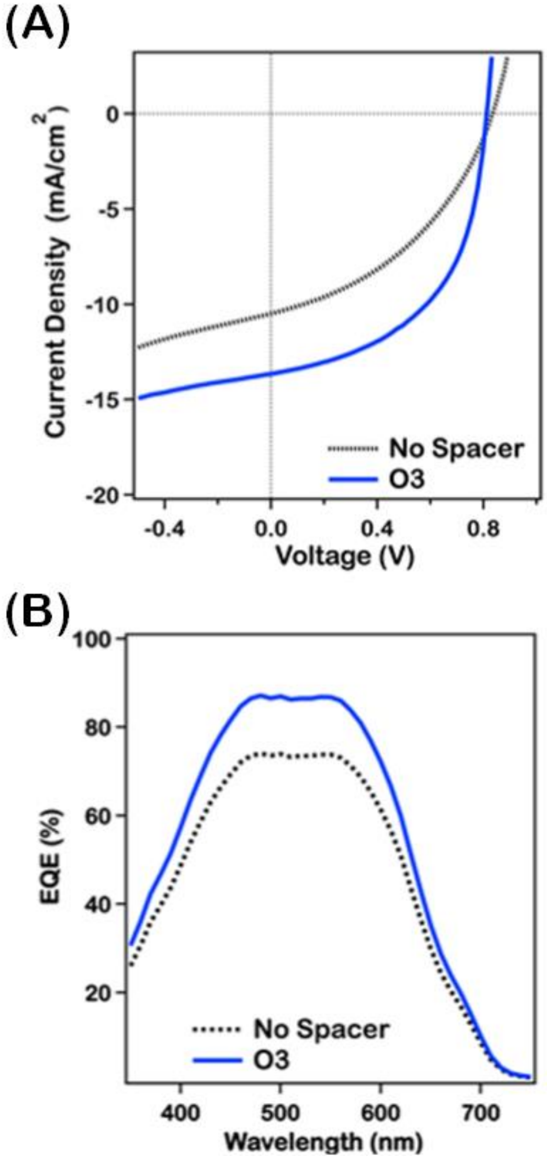

Fig. S12 (A) IV characteristics under AM 1.5 illumination and (B) external quantum efficiency for bulk-heterojunction solar cell with $PCDTBT:PC_{71}BM$ (1:4) as absorber without and with Oligothiophene O3 (6% by weight) modification

Another polymer system ($PCDTBT:PC_{71}BM$) is tested using O3 as spacer layer in bulk-heterojunction architecture. Using $PCDTBT:PC_{71}BM$ (1:4) with 6% O3 by weight, the J_{SC} increased from 12 mA/cm^2 up to 13.8 mA/cm^2.

14 Defining the role of spacer molecules in BHJ solar cells

A. Annealing experiment

We measured the photocurrent efficiency of a P3HT:O3:C60 device before (see Fig. S13 A) and after annealing (see Fig. S13 B). The blended device was prepared by mixing P3HT and O3(6 wt%, as used in the BHJ device in Fig 5 in manuscript) over night that resulted in a homogeneous mixture ensuring the incorporation of O3 in the bulk phase. The resulting solution was spin coated onto a ITO/PEDOT electrode and the device completed by thermally evaporating C60 followed by LiF(1nm)/Al (100nm). We also fabricated a P3HT/C60 bilayer device in the same method without O3 blending, which served as a control device. We measured the device performance for these devices before and after annealing and the results are illustrated in Fig.S13 C.

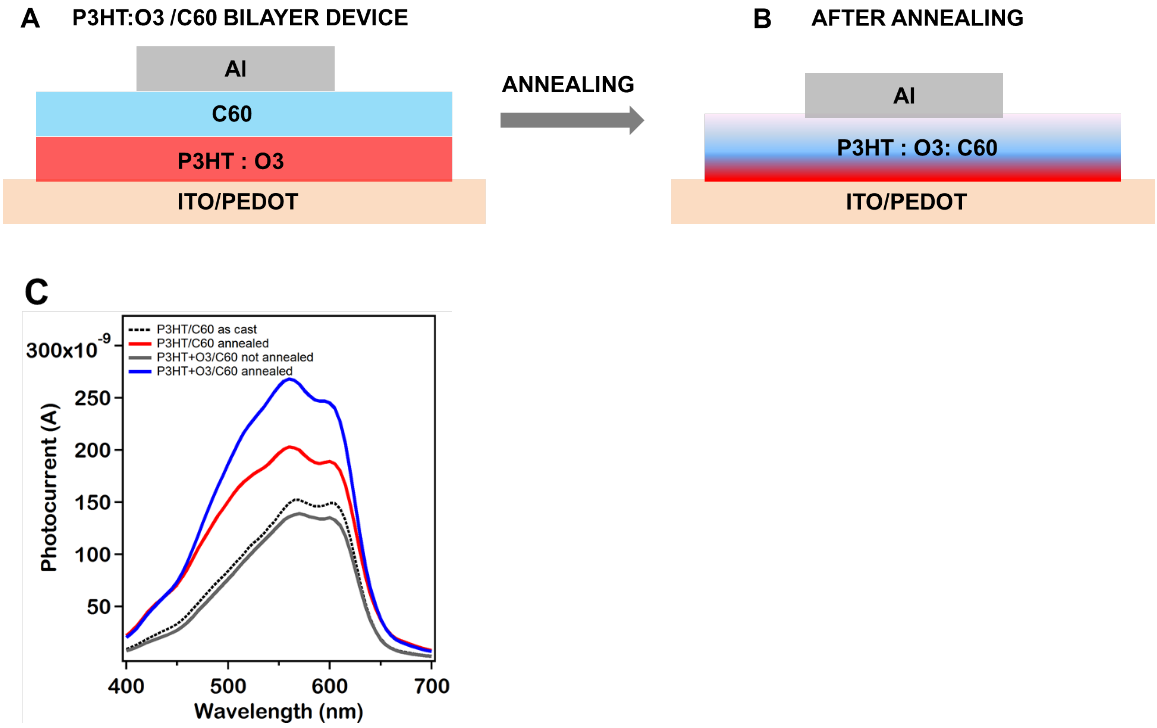

Figure S13: Describes the experiments to probe the role of O3 in charge separation at the donor/acceptor interface created by P3HT/C60.

No significant difference in photocurrent was observed between a P3HT/C60 and a P3HT+O3/C60 bilayer device. These results are in sharp contrast to our observation when O3 was precisely placed at the interface as shown in Fig. 1A of the manuscript, where a 350% increase in photocurrent was observed. These results clearly indicate that the presence of O3 at the donor/acceptor phase interface is critical and that it promotes extremely efficient charge separation and hence leads to a high photocurrent.

Next, we annealed both of these devices. The photocurrent increases as expected due to inter-diffusion of the donor and acceptor materials. However, the highest photocurrent was observed for devices with O3 indicating the crucial role played by the molecule. The increase in the photocurrent was ~60% in comparison to the device without O3, again consistent with the percentage increase we observed in the BHJ device in Fig.5 of the manuscript.

Although we cannot conclusively determine whether O3 undergoes phase separation, these measurements clearly show that, after thermal annealing, O3 plays a crucial role in promoting charge separation between the P3HT and C60, and the enhancement in the performance is due to the presence of O3 at the P3HT/C60 donor/acceptor interface. Furthermore, the trend observed as a function of loading volume % of O3 is consistent with that observed with the thickness dependence of the O3 in the bilayer devices (see Fig. 2 & Fig. 3 in the manuscript) again suggesting that the mechanisms for charge separation in the bilayer device with O3 at the interface or the BHJ device with O3 blended in are similar.

B impedance spectroscopy

Impedance spectroscopy is a powerful technique to probe interface charge transfer and recombination during device operation under continuous illumination and applied bias [10-12]. A typical BHJ device can be modeled using $R_s(R_1C_1(R_2C_2))$ circuit elements, where R_s is the contact resistance, R_1 corresponds to transport resistance in the bulk phase, C_1 represents the geometric capacitance for the device respectively; and R_2, C_2 describe the donor/acceptor interface property as shown in Fig. S14 A. Under illumination, the RC time constant $\boldsymbol{\tau}=R_2 \bullet C_2$ represents effective charge recombination lifetime.

Fig. S14 B shows a plot of the imaginary part of the impedance (Im(Z)) as a function of AC frequency for a no spacer device and devices with O3 (6%) and Irpiq (4%) spacers. These devices were particularly chosen as they demonstrate the highest increase in efficiency. The results show that the peak value of Im(Z) shifts to lower frequency upon incorporation of O3 and Irpiq. The change in time constant suggests a decrease of the interfacial recombination rate that leads to an increase in the efficiency of the devices. Such a phenomenon should not be observed, if O3 or Irpiq were present in the bulk phase. Also, the calculated effective interface recombination lifetime (the product of R_2 and C_2) increases and later gradually decreases with change in wt% of the O3 and Irpiq. We believe that at 6% loading for O3, the interface is saturated and further addition leads to its preferential incorporation in the bulk. All other parameters were constant, including the bulk resistance (R_1), the geometric capacitance (C_1)) and the contact resistance.

In conclusion, although the precise location of the O3 is not known, these results again clearly show that the photocurrent enhancement in our devices after incorporation of an O3 spacer layer

is due to its presence at the interface and not in bulk, and hence are depicted as shown in Fig. 5 of the manuscript.

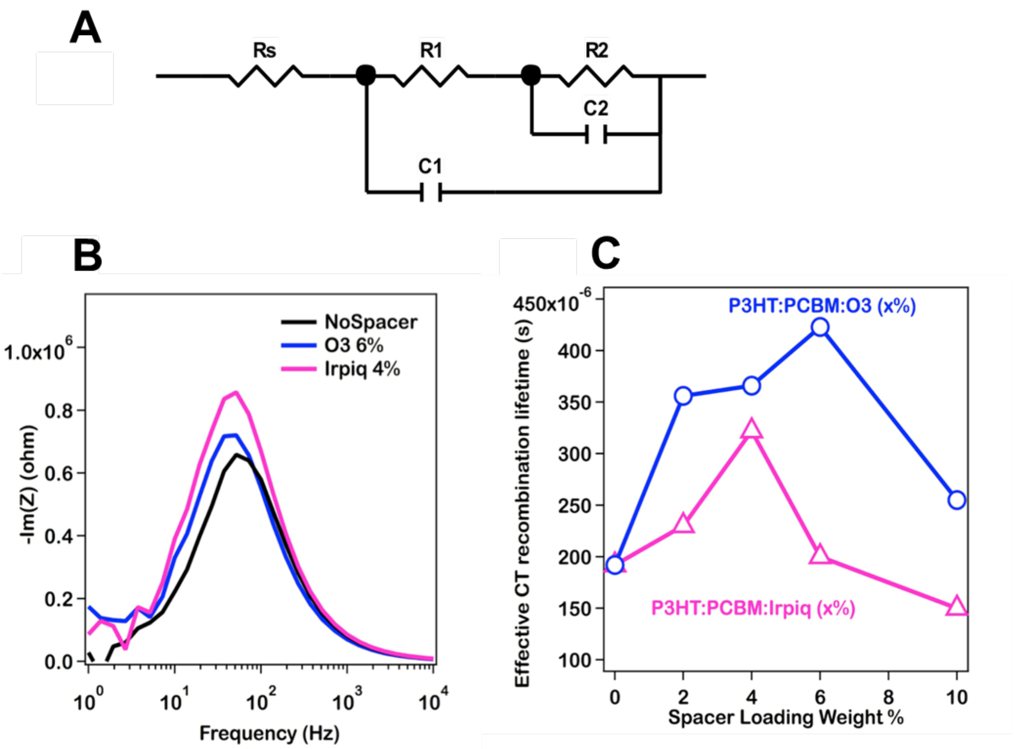

Figure S14: Describes the impedance measurements on BHJ devices with and without the spacer layers.

15 X-Ray diffraction on BHJ film without and with O3

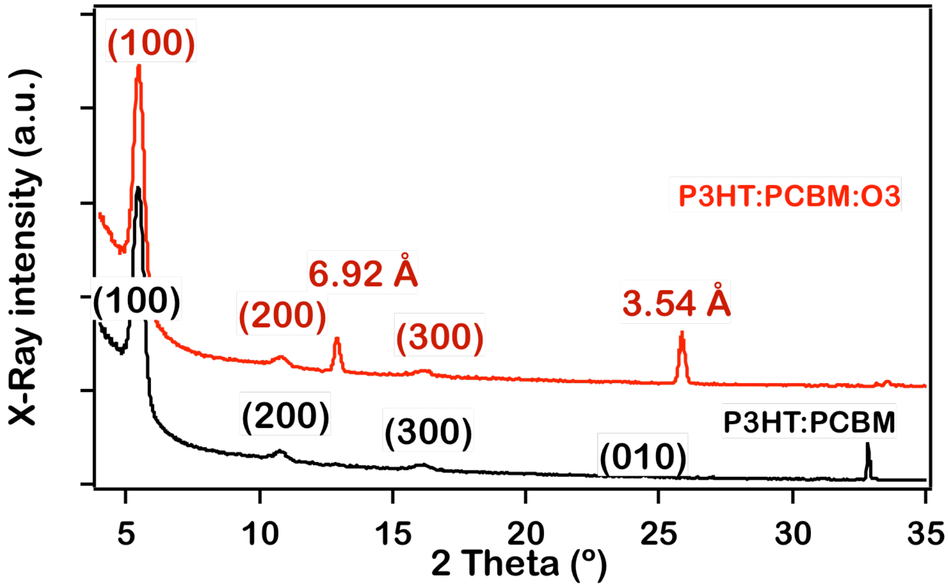

Figure S15 XRD spectrum for P3HT:PCBM blend film without and with O3 added. In both of the cases, the P3HT characteristic crystal peak at (100), (200) and (300) are identical, which indicates the addition of O3 didn't change the film morphology

15. Absolute absorption of bilayer device without/with LiF layer

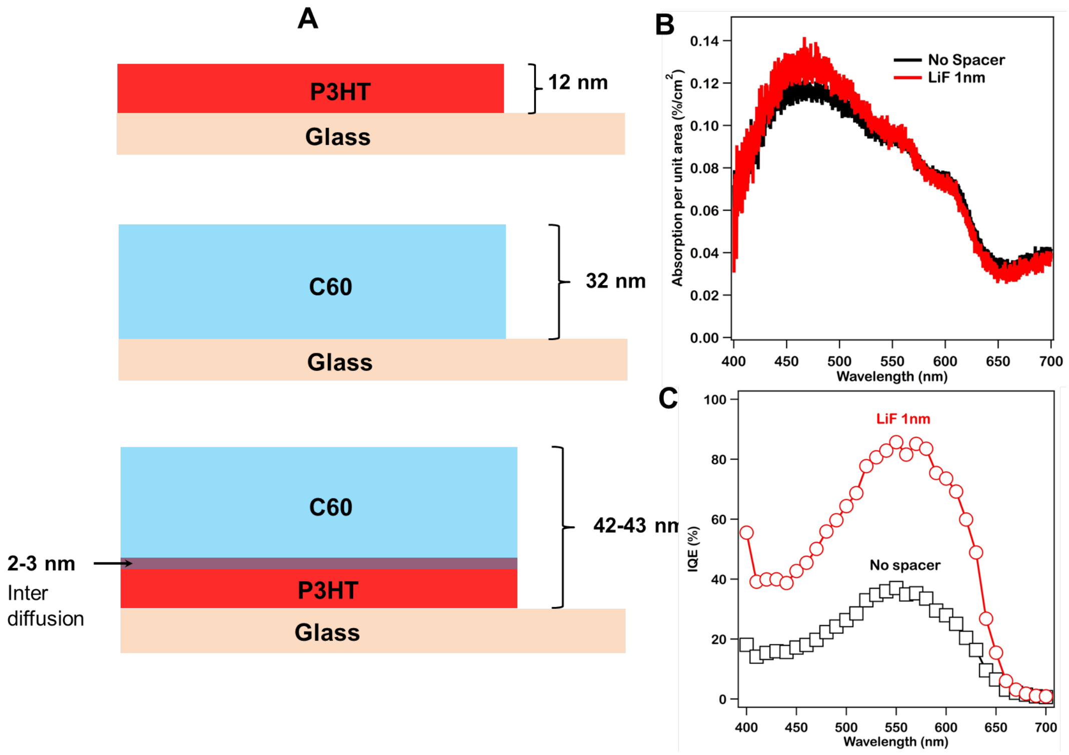

Figure S16: (A) Illustrates measurements to determine the inter-diffusion of C60 into the P3HT and (B) and (C) shows the absolute absorption and IQE measured using an integrating sphere for devices with and without a spacer layer respectively.

SI References

1. H. Meng et al., Oligofluorene−Thiophene Derivatives as High-Performance Semiconductors for Organic Thin Film Transistors. Chem. Mater. 15, 1778 (2003).
2. Jaramillo-Isaza, F & Turner, M. L. Synthesis and properties of conjugated oligomers containing fluorene, fluorenone, thiophene and cyclopentadithiophenone units. J. Mater. Chem. 16, 83 (2006).
3. Leever, B. J., Bailey, C. A. Marks, T. J. Hersam, M. C. & Durstock, M. F. In Situ Characterization of Lifetime and Morphology in Operating Bulk Heterojunction Organic Photovoltaic Devices by Impedance Spectroscopy. Adv. Energy Mater.2, 120 (2012).
4. Liu, F., Ruden, P. P., Campbell, I. H. & Smith, D. L. Device model for electronic processes at organic/organic interfaces. J. Appl. Phys. 111, 094507 (2012).
5. Liu, F., Crone, B. K., Ruden, P. P. & Smith, D. L. Control of interface microscopic processes in organic bilayer structures and their effect on photovoltaic device performance. J. Appl. Phys. 113, 044516 (2013).
6. Campbell, I. H. & Crone, B. K. Improving an organic photodiode by incorporating a tunnel barrier between the donor and acceptor layers. Appl. Phys. Lett. 101, 023301 (2012).
7. Kim, J. Y. et al., Efficient Tandem Polymer Solar Cells Fabricated by All-Solution Processing. Science 317, 222 (2007).
8. Zhang, Y. et al., Spin-enhanced organic bulk heterojunction photovoltaic solar cells. Nat. Commun. 3, 1043 (2012).
9. Rao, A. et al., The role of spin in the kinetic control of recombination in organic photovoltaics. Nature 500, 4 (2013).
10. G. Garcia-Belmonte et al., Charge carrier mobility and lifetime of organic bulk heterojunctions analyzed by impedance spectroscopy. Organic Electronics 9, 847 (2008)
11. B. J. Leever, C. A. Bailey, T. J. Marks, M. C. Hersam, M. F. Durstock, In Situ Characterization of Lifetime and Morphology in Operating Bulk Heterojunction Organic Photovoltaic Devices by Impedance Spectroscopy. Advanced Energy Materials 2, 120 (2012)
12. W. L. Leong, S. R. Cowan, A. J. Heeger, Differential Resistance Analysis of Charge Carrier Losses in Organic Bulk Heterojunction Solar Cells: Observing the Transition from Bimolecular to Trap-Assisted Recombination and Quantifying the Order of Recombination. Advanced Energy Materials 1, 517 (2011)
